# A Brief Overview of Preeclampsia

**DOI:** 10.4021/jocmr1682w

**Published:** 2013-12-13

**Authors:** Noura Al-Jameil, Farah Aziz Khan, Mohammad Fareed Khan, Hajera Tabassum

**Affiliations:** a Department of Clinical Laboratory Sciences, College of Applied Medical Sciences, King Saud University, Riyadh, Kingdom of Saudi Arabia

**Keywords:** Preeclampsia, Eclampsia, Hypertension, Proteinuria, Microangiopathy

## Abstract

Preeclampsia (PE) is a leading cause of maternal mortality and morbidity worldwide. It occurs in women with first or multiple pregnancies and is characterized by new onset hypertension and proteinuria. Improper placentation is mainly responsible for the disease. If PE remains untreated, it moves towards more serious condition known as eclampsia. Hypertension, diabetes mellitus, proteinuria, obesity, family history, nulliparity, multiple pregnancies and thrombotic vascular disease contribute as the risk factors for PE. PE triggered metabolic stress causes vascular injury, thus contributing to the development of cardiovascular disease (CVD) and/or chronic kidney disease (CKD) in future. This risk appears to be increased especially in women with a history of recurrent PE and eclampsia. Clinically increased serum levels of sFlt-1 and decreased placental growth factor (PIGF) and vascular endothelial growth factor (VEGF) represent the severe condition of PE. The clinical findings of sever PE are assorted by the presence of systemic endothelial dysfunction, microangiopathy, the liver (hemolysis, elevated liver function tests and low platelet count, namely HELLP syndrome) and the kidney (proteinuria). The early detection of PE is one of the most important goals in obstetrics.

## Introduction

Preeclampsia (PE) is a most frequently encountered renal complication of pregnancy and it is characterized by hypertension, proteinuria and edema, usually by the last trimester of pregnancy. The rate of incidence varies upon the study population but generally ranges from 3% to 7% of all pregnancies [1-4]. It occurs manly in the women in their first pregnancies or to those who carry twins [5]. PE when remains untreated, it moves towards more serious condition known as eclampsia, and is still one of the leading causes of maternal and neonatal mortality. It takes place only in the presence of placenta even without fetus (hydatidiform mole), and typically improves postpartum [6, 7]. Hypoperfusion and ischemic conditions evidently show the abnormal placenta. PE is known to be originated from disordered vascular development of the placenta which further widely spreads anti-angiogenic factors into the maternal circulation and causes a systemic endothelial cell dysfunction and microangiopathy [8, 9]. Upon kidneys these endothelial damages result in glomerular endotheliosis and proteinuria in which the endothelial cells of the glomerulus swell and endothelial fenestrations are lost [1, 10].

As mentioned above, the untreated complication of PE is eclampsia. It is defined by the presence of seizures for which women are often treated with magnesium sulfate prophylaxis [11]. The clinical findings of sever PE are assorted by the presence of systemic endothelial dysfunction and microangiopathy, the liver (hemolysis, elevated liver function tests and low platelet count, namely HELLP syndrome) and the kidney (proteinuria) [12].

## Risk Factors and Pathogenesis of Preeclampsia

PE is a disorder of pregnancy which affects various organs. Hypertension, diabetes mellitus, proteinuria, obesity, family history, nulliparity, multiple pregnancies, use of contraceptions, older women conception (> 40) and thrombotic vascular disease contribute as the risk factors for PE [13, 14]. New onset of hypertension (systolic ≥ 140 mmHg in 24 h urine) can be seen after 20 weeks of gestation in previously normal women. Several other symptoms like edema, renal failure, liver failure and HELLP syndrome also worsen the clinical condition [15]. Depending upon the early or late onset, disease can show mild or severe symptoms. Deranged liver function, thrombocytopenia and HELLP syndrome can evolve in eclampsia in severe cases [16, 17].

Nulliparous women (who conceived through assisted reproductive techniques) and women already affected by autoimmune disease can experience disturbed maternal immune system in the early onset [18, 19]. On the contrary, women with metabolic, renal or vascular disease are especially at increased risk for superimposed PE. Environmental factors may also contribute to the disease for those who are living at higher altitude and facing hypoxic conditions [20].

In a study, Cunningham et al evaluated 37 pregnant women and reported 58% and 64% PE in subjects with moderate and severe renal insufficiency respectively [21]. On the contrary, Fink et al observed a ratio of 7.3 for PE in 169 women with renal disease [22]. The histopathological origin widely differs but diabetic nephropathy is known to be the most common cause of chronic kidney disease (CKD) in pregnancy [22].

The abnormal placenta is known to be the major cause for the origin of PE, and its removal puts an end to the disease. Due to abnormal implantation and placentation poor uterine and placental perfusion occurs which leads to oxidative stress, hypoxic condition and release of some anti-angiogenic factors [23]. These anti-angiogenic factors lead to generalized endothelial dysfunction which is responsible for hypertensive syndrome and microangiopathy [23]. Pathological examination of placentas from women with severe PE reveals several abnormalities like infarcts, thrombosis, atherosis and chronic inflammation [24]. Some anti-angiogenic proteins produced by abnormal placenta are soluble endoglin (sEng) and the soluble vascular endothelial growth receptor-1 (sFlt-1), which induce endothelial dysfunction by inhibiting pro-angiogenic factors like placental growth factor (PIGF) and vascular endothelial growth factor (VEGF) [25-28]. Maternal blood levels of sFlt-1 represent the severity of PE whereas oppositely quantities of VEGF and PIGF were decreased in patients with severe symptoms when compared to normal pregnancies. Alterations in sFlt-1 and PIGF are also more pronounced in early onset in comparison to late onset PE [29]. Before going to discuss the role of anti-angiogenic factors, we put a light over the role of pro-angiogenic growth factors and their receptors in vascular homeostasis.

### Pro-angiogenic factors

VEGFs are dimeric glycoproteins involved in angiogenesis and vasculogenesis [30]. VEGF family receptors present on vascular endothelial cells include Flt-1 (VEGFR-1) and KDR (VEGFR-2). VEGFs are known to bind with both kinds of receptors, namely Flt-1 and KDR, while PIGF homodimers bind specifically to Flt-1. VEGFs mainly act upon VEGFR-2 to affect endothelial cells [31]. Chappel et al suggest that the role of Flt-1 gene to express sFlt-1 which acts by regulating guidance of emerging vessel sprouts by modulating local VEGF availability [32]. The extirpation of single VEGF allele results in markedly abnormal vasculature inkling the placental vasculature with death at embryonic days 10-12 [33]. The survival of endothelial cells and vascular homeostasis in mature vessels and tissues is because of VEGF. Cells responsible for VEGF expression are located adjacent to fenestrated endothelia, epithelial cells of the choroid plexus, renal podocytes and hepatocytes [34, 35]. The inhibition of VEGF leads to pathological conditions in many of the organs with fenestrated endothelia and it is also observed in PE. VEGFs have a direct vasodilatory effect on the systemic vasculature because infusion of VEGF leads to nitric oxide-dependent vasorelaxation in the coronary artery [36].

PIGF shows similarity to VEGF and expresses at high levels from placenta. As mentioned earlier, it binds with Flt-1 receptor with high affinity. In an animal study PIGF null mice exhibit defects in tumor angiogenesis, myocardial neovascularization and wound healing suggesting that PIGF plays a role in angiogenesis in pathological settings [37]. PIGF binds itself more actively with Flt-1 receptor by displacing VEGF from Flt-1; other possible mechanisms include direct effects of Flt-1 signaling and the formation of VEGF/PIGF heterodimers. During pregnancy placenta releases high quantity of PIGF and their levels increase from second trimester and thereafter decline [38].

### Anti-angiogenic factors

Various studies have potentially established the presence of factors in the circulation of preeclamptic women released by injured or activated endothelium. Among other these factors include endothelin-1, fibronectin, von Willebrand factor, oxidative stress markers, thrombomodulin and inflammatory cytokines [39]. There is a deficiency of prostacyclin, nitric oxide, vasodilatory factors in the circulation of women with PE [40]. A theory, that circulating factors cause the endothelial dysfunction, was strengthened when *in vitro* serum from women with preeclampsia induces endothelial dysfunction and injury [41].

Anti-angiogenic factors release from the placenta and enter into maternal circulation, which is the main cause of endothelial dysfunction in PE. An anti-angiogenic protein, soluble fms-like tyrosine kinase (sFlt-1, also known as sVEGFR-1) is a soluble form of VEGF/PIGF receptor Flt-1 produced by alternative splicing [42]. sFlt-1 inhibits VEGF and PIGF activity by halting endothelial tube formation and blocking their vasodilatory effect. In an animal study administration of sFlt-1 to pregnant rats induces hypertension, proteinuria and glomerular endotheliosis [43].

Another anti-angiogenic factor is sEng found from placentas of women with PE. Studies have identified sEng levels 4-fold higher in females with severe PE than normal women. sEng when combined with sFlt-1 induces features of sever PE like liver dysfunction, restricted fetal growth, coagulation and neurological abnormalities [44, 45]. In an animal study, sEng stricts the formation of endothelial tube and increases capillary permeability in mouse, liver, lung and kidney. When pregnant rats were dozed with combined sFlt-1 and sEng marked features of PE were developed, namely hypertension, nephrotic syndrome, low platelet count, elevated liver enzymes and reduced fetal weight [44].

## Pathophysiology of Various Clinical Instances

### Hypertension

Hypertension is very commonly seen in PE. Increased peripheral vascular resistance rather than increased cardiac output is the cause of hypertension [46]. PE exaggerates response to angiotensin II, catecholamines and other hypertensive stimuli when compared to normal controls [47]. A study has reported that the effect may elevate the onset of overt hypertension by weeks to months [48]. In PE total plasma volume gets low [49] and probably increases circulating volume as evidenced by suppressed rennin and aldosterone [50, 51] and elevated natriuretic hormone compared to normal pregnancy [52]. The markers of endothelial activation and dysfunction like endothelin, cellular fibronectin, plasminogen activator inhibitor-1 (PAI-1) and von Willebrands factor are altered in PE. In *in vitro* studies altered endothelial functions have been observed in preeclamptic vessels [53, 54]. The increased incidence of PE in women with chronic diseases like diabetes and hypertension suggests some factor in maternal milieu may also lend susceptibility to PE. Several mediators like vasoconstrictors norepinephrine, endothelin, thromboxane and vasodilators prostacyclins and nitric oxide are known to mediate vasoconstriction.

### Cardiovascular risk

PE can proceed to permanent vascular, metabolic damage and also elevate the risk of cardiovascular disease (CVD). A study showed increased relative risk of CVD after PE [55]. CVD is known to be the major cause of mortality worldwide [53]. In a study insulin resistance and angiogenesis dysfunction revealed the association between PE and metabolic syndrome which showed even one year after delivery serum glucose and sFlt-1 continuously increase [56]. It is noted that increased rates of CVD and type 2 diabetes were observed in gestational hypertension. Large retrospective epidemiological studies have consistently demonstrated elevated risk for many types of CVD in women with history of PE. CVD and PE have some common risk factors like obesity and metabolic syndrome. Therefore as PE is known to be the risk factor for CVD, CVD does the same with PE [57-61]. Women had PE before 34 weeks or PE combined with preterm birth has an even higher risk of death from CVD, at four to eight times the risk of women who had a normal pregnancy [62].

Persons born to pregnancies complicated by PE or gestational hypertension may also have some future cardiovascular risks. Increased risk of hemorrhage and ischemic stroke after the stages of 60 - 70 years was reported, mainly among those with the history of severe PE, maybe due to cerebral vascular dysfunction brought about by development failure during intrauterine life [63-65].

### Hyperuricemia

More than 80 years before the relation between serum uric acid and PE has been established [66]. Serum uric acid levels are proportional in women with PE, severity of proteinuria, renal changes, maternal morbidity and fetal demise [67, 68]. In a recent study, it has been suggested that hyperuricimea directly contributes to vascular damage and hypertension [69].

### Neurological abnormalities

If PE remains untreated, the condition is complicated by the development of seizures in brain and is referred as eclampsia. Headache, blurred vision and temporary loss of vision are few symptoms of eclampsia. The neurological changes like cerebral edema and vasoconstriction have been attributed in PE. The cerebral edema involves the posterior portions of the white matter and known as reversible posterior leuko encephalopathy syndrome (RPLS) [70, 71].

## Renal Effects of Preeclampsia

The kidneys are among the main organs affected by PE and result can be seen in the form of proteinuria. The level of proteinuria is directly related to poor maternal and prenatal prognosis and higher risk of complications like eclampsia and HELLP syndrome. The urine protein to creatinine ratio (P:C ratio) has become the preferred method to quantify proteinuria in non-pregnant population but it is still controversial in the diagnosis of PE [72, 73]. Microalbuminuria is a marker of renal endothelial injury resulting from local or systemic vascular damage. There is an increased risk of persistent microalbuminuria after PE and this may be the main cause of kidney disease after PE [74, 75]. In a study who previously encountered PE have increased risk of later end stage renal disease, and women with recurrent preeclamptic pregnancies and who give birth to offspring with low birth weight had even high risk. Many mechanisms explain the association between PE and renal disease. Hypertension, endothelial dysfunction, obesity and CVD are the risk factors for chronic kidney disease and PE seems to be related four to five times higher related risk with CKD and microalbuminuria [76, 77].

The glomerular consequences of PE can be understood in terms of disruption of glomerular filtration barrier (GFB) through glomerular endothelial cell injury. Not only endothelial damage results in endotheliosis, the podocytes are simultaneously disrupted, since these specialized cells are highly dependent on signals derived from glomerular endothelial cells to maintain structures and the diaphragms [78]. Glomerular endothelial cell injury causes the breakdown of multiple components of GFB which leads to proteinuria and hypertension. Arteriolar endothelial injury also occurs in PE and may induce narrowing of arteriolar lumina. Thus, glomerular filtration is compromised and manifests as elevated blood pressure [79].

The decreased GFR is because of endotheliosis and primarily through reduction in the ultra filtration coefficient as opposed to diminished plasma flow. Mild forms have been observed in 30% patients with pregnancy-induced hypertension without proteinuria [80, 81]. A recent study found 5 of 12 control subjects with trace endotheliosis. Limited endotheliosis have also been reported occasionally in association with other disorders [82]. Studies revealed that women with PE history have elevated blood pressure, endothelial dysfunction, weight gain and other signs of adverse cardiovascular risk profile both before and after preeclaamptic pregnancy [83, 84]. No study has suggested that preeclampsia itself has negative effect on blood pressure and microalbuminuria although studies suggest its effect on worsening cardiovascular risk profile after pregnancy in women with PE. It is also noted that extensive glomerular changes can also be resolved postpartum. The fact that 20-40% of women have microalbuminuria after preeclamptic pregnancy is in confusion for a permanent glomerular damage in a great proportion of these women. Several investigators argue that proteinuria itself causes continued renal dysfunction via increased interstitial inflammation, and same for microalbuminuria [58].

## Conclusion

Preeclampsia is a systemic disorder characterized by maternal endothelial dysfunction. Adequate screening, monitoring and routine check-up during and after pregnancy may prevent worsening the maternal and fetus condition. Previous history of PE helps with the assessment of early diagnosis and the risk of CVD and CKD. This review summarizes the systemic endothelial dysfunction, risk factors, pathogenesis, pathophysiology and renal effects of PE.
